# Measuring and Validating a General Cancer Predisposition Perception Scale: An Adaptation of the Revised-IPQ-Genetic Predisposition Scale

**DOI:** 10.1371/journal.pone.0142620

**Published:** 2015-11-11

**Authors:** Wendy Wing Tak Lam, Qiuyan Liao, Jennifer Hiu Fai Wong, Ching Lung Lai, Man Fung Yuen, Janice Wing Hang Tsang, Richard Fielding

**Affiliations:** 1 School of Public Health, The University of Hong Kong, Hong Kong Special Administrative Region, People’s Republic of China; 2 Department of Medicine, The University of Hong Kong, Hong Kong Special Administrative Region, People’s Republic of China; 3 Department of Medical Oncology, The University of Hong Kong, Hong Kong Special Administrative Region, People’s Republic of China; Taipei Medical University, TAIWAN

## Abstract

**Background:**

Illness perceptions are linked to individual help-seeking and preventive behaviors. Previous illness perception studies have identified five dimensions of illness-related experience and behaviour. The Revised Illness Perception Questionnaire (IPQ-R) for genetic predisposition (IPQ-R-GP) was developed to measure illness perceptions in those genetically-predisposed to blood disease. We adapted the IPQ-R-GP to measure perceptions of generalized cancer predisposition. This paper describes the development and validation of the Cancer Predisposition Perception Scale (CPPS).

**Methods:**

The draft CPPS scale was first administered to 167 well Hepatitis B carriers and 123 other healthy individuals and the factor structure was examined using Exploratory Factor Analysis. Then the factor structure was confirmed in a second sample comprising 148 healthy controls, 150 smokers and 152 passive smokers using Confirmatory Factor Analysis (CFA).

**Results:**

Six-factors comprising 26 items provided optimal fit by eigen and scree-plot methods, accounting for 58.9% of the total variance. CFA indicated good fit of the six-factor model after further excluding three items. The six factors, Emotional representation (5 items), Illness coherence (4 items), Treatment control (3 items), Consequences (5 items), Internal locus of control (2 items) and External locus of control (4 items) demonstrated adequate-to-good subscale internal consistency (Cronbach’s α = 0.63–0.90). Divergent validity was suggested by low correlations with optimism, self-efficacy, and scales for measuring physical and psychological health symptoms.

**Conclusion:**

The CPPS appears to be a valid measure of perceived predisposition to generic cancer risks and can be used to examine cancer-risk-related cognitions in individuals at higher and lower cancer risk.

## Introduction

Illness perceptions reflect cognitive-affective representations of the illness characteristics, causes, trajectories, consequences and associated impacts [1]. Illness perception includes the self-evaluation by an individual of the risks of developing a specific illness at some point in life [1]. Knowledge of individual illness perceptions can inform strategies to raise prevention awareness or assistance for support and adaptation to the illness in question. In the context of cancer, primary and secondary prevention remain the most effective control strategies. These range from population-level interventions, such as smoking restrictions and cessation, recognition and prompt presentation of suspicious symptoms, through to clinical interventions such as surgery and chemotherapy. In cancer early diagnosis and prompt treatment are crucial to survival. In this regard, awareness of potential risk can facilitate preventive behaviours, adherence to appropriate screening [2] and prompt presentation of symptoms [3]. Hepatocellular carcinoma (HCC, or primary liver cancer) is the third most common cause of cancer death globally. A disproportionately large percentage of the HCC deaths are in the Asia-Pacific region [4]. East and South-East Asia account for 75% of global hepatitis B virus (HBV) carriage [5]. Hepatitis B surface antigen (HBsAg) carriers comprise the highest-risk group for HCC. However, it is tobacco use that accounts by far for the largest number of cancer cases, importantly those of the respiratory system, which are prominent wherever smoking has been established for several decades [6]. It is estimated that lung cancer risk is 23.6 times higher in male current smokers and 7.8 times higher in female current smokers relative to never smokers [7] while never-smokers who live with a smoker (passive smokers) have 1.19 times the lung cancer risk relative to those not living with a smoker [8]. Little is known about the associations between illness perception and perceived cancer risk within different risk groups such as HBsAg carriers, active smokers and passive smokers. As a first step, a suitable tool is needed.

Self-regulation theory postulates that people develop goals, towards which their thinking and behavior are then directed at achieving [9]. Leventhal, et al. argue that risk perception is the primary determinant of how an individual copes with health threats cognitively and behaviourally in order to attain a goal, such as staying healthy [10]. The Illness Perception Questionnaire (IPQ) was developed in light of self-regulation theory to measure individuals’ illness perceptions. However, the original IPQ neglected emotional representation components while the cure/control and timeline components failed to factor completely into their respective domains [11]. The IPQ-R was therefore developed to remedy shortcomings in the original scale [12].

Subsequently, the IPQ-R has been validated for use in several different diseases or health groups [1, 13–18]. However the IPQ was not primarily developed to assess perceived cancer predisposition, and though adapted to look at predisposition to genetic disease risk, it has not yet been adapted to look at general cancer predisposition. This paper reports the preliminary development, reliability and validity of an instrument derived from the IPQ to measure perceived cancer predisposition, namely Cancer Predisposition Perception Scale (CPPS).

## Methods

### Participants

The study was conducted in two stages.

#### Stage I

Two groups were recruited in Stage I for item reduction and to explore the factor structure of the draft CPPS: Hepatitis B virus positive clinic attendees (“HBV group”) and healthy community-dwelling adults (“Non-patient group”). The HBV group consisted of asymptomatic Chinese men and women with known HBsAg carrier status who are otherwise healthy. Between April 2011 and March 2013 clinical staff from the relevant out-patient department of a Hong Kong Hospital identified HBV patients after their follow-up appointments. Eligible HBV subjects had no known advanced liver disease such as cirrhosis or HCC, related indicators thereof, or other co-morbid chronic disease. Exclusion criteria for both groups were those who are (a) unable to understand and communicate in Cantonese, (b) had hearing deficits, (c) intellectual deficits, or (d) Axis I psychiatric diagnoses. Eligible HBV patients were approached prior to their consultation at a local University teaching hospital out-patient clinic by trained interviewers. Adult friends and family accompanying the patients were recruited as participants for the "Non-patient" group in the study at this time if they were willing and gave informed consent. Other "Non-patient" group subjects were recruited from adult family and friends of staff employed by the University of Hong Kong. All interviews were conducted by a trained research assistant. Participants self-completed the questionnaires, unless sub-literacy dictated a face-to-face oral interview. While mixing completion methods raises the possibility of bias, exclusion of sub-literate but eligible subjects clearly increases it. Comparison of self-completing questionnaires with interview completion indicate no significant differences [19]. Subsequent comparisons of scores showed no systematic bias attributable to completion style.

#### Stage II

Three groups were recruited in Stage II for testing the validity of the constructs derived in Stage I using Confirmatory Factor Analysis (CFA). These included adults (aged≥18) who currently smoked cigarettes (current smokers), or who were never smokers living with at least one current smoker who smoked at home (passive smokers) or who were never smokers not living with current smokers (healthy controls). All participants, who were required to be currently healthy with no history of significant illness, were recruited using random digital household telephone interview based on landline. It is reported that landline penetration is approximate to 100% in Hong Kong [20]. To screen for eligible subjects, the first-contact person of the selected household was asked if any adults who were currently smokers lived in the household. For households with at least one current smoker, one current smoker and one passive smoker of that household who fulfilled the inclusion criteria were invited to participate in the telephone interview. For households without current smokers, one adult whose birthday was most proximal to the survey date was invited to participate. All interviews were conducted by trained interviewers during non-working hours (6:00–10:00 pm) between October and November 2012.

### Ethics statement

The study obtained ethical approval from the Institutional Review Board of the University of Hong Kong/Hospital Authority Hong Kong West Cluster. In Stage I, all eligible participants (the “HBV group” and “Non-patient group”) gave written informed consent after being fully informed about the purpose of the study, data confidentially and rights to refusal and uncontested withdrawal. In Stage II, since subjects (current smokers, passive smokers and healthy controls) were recruited using telephone interview, written informed consent could not be obtained and thereby was waived by the ethics committees. Therefore, all subjects who were willing to participate in the study in Stage II gave oral consent before they answered the questionnaire.

### Instruments

All participants completed the CPPS, along with five comparative scales included to evaluate validity.

#### Item derivation

The original IPQ-R demonstrates higher internal consistency (Cronbach alphas range from 0.75 to 0.89) than the original IPQ and good test-retest reliability ranging from 0.46 to 0.88 over three weeks [12]. From among the different existent versions of the IPQ-R, the version for genetic predisposition (IPQ-R GP) to venous thrombosis (genetic mutation in Factor V-Leiden) [1] most closely approximated to our research focus. The IPQ-R GP has satisfactory internal reliability, ranging from 0.69 to 0.80 but low internal consistency for the “treatment control” subscale, which the developers were unable to improve [1]. We adapted the IPQ-R-GP by modifying the scale focus from genetic mutation to cancer predisposition by re-wording items accordingly but retaining wherever possible the item focus, to produce a draft questionnaire. We excluded symptoms and time-line subscales from the original IPQ finally retaining 28 items. The scale measures five components of illness perception: emotional representation, personal control, treatment control, perceived consequences, and illness coherence, tailored to cancer predisposition. We avoided using the IPQ appellation in relation to our instrument out of respect for the IPQ developers as this was an unofficial adaptation but acknowledge its contribution.

#### CPPS

The draft CPPS comprised 28 items translated from the IPQ-R GP which were re-worded in English by one of the authors to address general cancer predisposition. Retaining item focus, positive/negative phrasing and scoring direction was done to cover the original five IPQ domains: emotional representations, illness coherence, perceived consequences, personal control and treatment control, while retaining a balance of positive and negatively worded items. We excluded the sections on the causes of predisposition and the open-ended questions from the IPQ-R GP as being too varied for this focus. Following standard procedure the draft instrument was translated into Chinese by one bi-lingual team member and then back-translated into English by a second independent professional translator. Original and back-translated versions were compared and amended as necessary and translation procedures reiterated until a consensus was reached and the meaning was equivalent to the original version. Participants indicated the extent to which they agree or disagree with the statements concerning their perception of their cancer predisposition, using a 5-point Likert scale: 1-Strongly disagree; 2-Disagree; 3-Neither agree nor disagree; 4-Agree; 5-Strongly agree.

#### Additional measures

Participants also completed the following validated Chinese language measures:

Perceived General Health (PGH) and Perceived Current Health (PCH) were each measured on a single four point Likert scale, 1 = very good, 2 = fair, 3 = poor, 4 = very poor. Perceived health is a simple but powerful and consistent predictor of future health state, health care utilization and mortality, widely used as a valid perceived health indicator [21, 22]. Both PGH and PCH were reverse coded so that higher PGH and PCH scores indicate better perceived health.

The revised Chinese Life Orientation Test (C-LOT-R) is a measure of outcome orientation (dispositional optimism-pessimism) [23]. The scale comprises 6 items measuring the underlying dimensions of optimism and pessimism involving three positively-worded and three negatively worded statements scored on a 4-point Likert scale. Participants indicated agreement with each statement from ‘Completely disagree’ to ‘Completely agree’. The instrument has good psychometric properties in the Hong Kong and other Chinese communities. The negatively-worded statements were reversely coded and the total score of all 6 items were calculated. Higher total CLOT-R scores indicate higher dispositional optimism.

The General Self-Efficacy Scale (GSES) is a 10-item measure of generalized self-beliefs about personal competency when dealing with difficult situations [24]. The GSES is scored on a 4-point Likert scale, with options ranging from ‘Completely incorrect’ to ‘Completely correct’ to indicate the degree to which participants agree to the statements. The GSES has been validated for use in the Hong Kong Chinese community (CGSES) and has good psychometric properties (Cronbach’s alpha = 0.92) [25]. A total score of CGSES was calculated with higher total scores indicating greater perceived generalized self-efficacy.

The Chinese Health Questionnaire-12 (CHQ-12) is a shortened form of Goldberg’s General Health Questionnaire adapted for the Chinese population [26]. Developed to screen for symptoms of psychological morbidity in the general community dwelling population the 12-item CHQ-12 measures general somatic and psychological symptoms associated with anxiety and depression. Participants indicated agreement with each statement on a 4-point Likert scale ranging from ‘Not at all’ to ‘Much more than usual’. Higher scores indicate greater psychological symptomatology. Having a Cronbach alpha = 0.8, the CHQ-12 has been extensively validated for use in Chinese communities [26, 27].

#### Demographics

Demographic and medical data were collected for validation of diagnosis (where appropriate) and for comparative purposes. The information was either provided by the participants or obtained from medical records.

### Statistical analysis

In Stage I, exploratory factor analysis (EFA) using principal components analysis with direct oblimin rotation of factors was used to optimize the factor structure, using eigenvalues >1.0 and scree-plot criteria. A five-factor structure was anticipated, as proposed by the existing model but alternative solutions either side of this were examined for better data fit. Bartlett’s test of sphericity and the Kaiser-Meyer-Olkin index of sampling adequacy were used to determine the sample appropriateness for factor analysis. The internal consistency of the scale was calculated by Cronbach’s alpha coefficient and was deemed acceptable if α≥0.7. The draft instrument scores were compared against the PGH, PCH, C-LOT-R, CGSES, and CHQ-12 scales to assess divergent validity. We hypothesized that the CPPS would not highly correlate with these scales. Construct validity was evaluated using known group approach, comparing each CPPS subscale score for HBsAg carriers against those of the non-patient group. All analyses were performed with SPSS version 19 for Windows.

In Stage II, the factor structure derived from Stage I was tested using CFA. All factor loadings and measurement errors of the indicators were simultaneously estimated in CFA. Multiple model fit indices including χ^2^/d_f_, CFI, TLI, RMSEA and SRMR were used to evaluate the model fit. A model with values of χ^2^/d_f_<3, CFI>0.90, TLI>0.90, RMSEA<0.08 and SRMR<0.05 was considered to be acceptable [28]. If fit indices indicated model mis-specification, the model was re-specified by examining the factor loadings and model modification indices. Indicators with very low factor loadings (λ<0.3) were removed and the CFA re-run. The CFA was performed using Mplus 6.0. Then scores of the confirmed subscales of the draft CPPS in current smokers and passive smokers were compared against those of the healthy controls to further assess construct validity using t-tests. P-values<0.025 were considered statistically significant after Bonferroni correction.

## Results

### Participants

A total of 290 participants completed the questionnaire in Stage I, 167 of whom were HBsAg carriers, while 123 were non-patients. Demographic variables, including age, sex, marital status, education level and occupation indicated that the non-patients were on average younger than the HBV patients and included more female, single and higher educated members compared to the HBsAg group (S1 Table).

A total of 150 current smokers, 152 passive smokers and 148 non-smoking healthy adults were recruited in Stage II. The three samples differed significantly in gender, age, education level and occupation (p<0.05). Current smokers were more likely to be male with lower educational attainment in full-time employment while healthy controls were more likely to be older and have higher educational attainment. The characteristics of the participants are presented in S2 Table.

### Stage I

#### EFA

In contrast to the parent IPQ-R(GP) [1], EFA-derived eigenvalues and scree plots indicated a six-factor structure provided the best fit to the data giving maximum item loading and minimal cross-loading. The oblique (direct oblimin) rotation was used to generate further loadings. Item loadings below λ < 0.40 were suppressed. One of the draft items ‘My predisposition does not worry me’, originally under the emotional domain, failed to load onto any factor and was removed and the EFA was reiterated. Another item "There is a lot which I can do to control the risk situations that predispose to cancer" did not load highly (λ<0.40) on any of the six resulted factors was also excluded. A final six-factor model comprising 26 items accounted for 60.5% of total variance. The six factors are named "Emotional representation" (5 items), "Illness coherence" (5 items), "Treatment control" (3 items), "Consequences" (6 items), "Internal control" (3 items) and "External control" (5 items). The factor loadings of all selected indicators on their corresponding factors are shown in Table 1.

**Table 1:** Item factor loadings in exploratory factor analysis in Stage I.

Item						
	Emotional Representation	Illness Coherence	Treatment Control	Consequences	Internal Control	External Control
I25. Having a possible predisposition to cancer makes me anxious	0.88					
I26. To be possibly predisposed to cancer makes me afraid	0.85					
I22. I get depressed when I think about my possible predisposition to cancer	0.83					
I23. When I think about my possible predisposition to cancer I get upset	0.80					
I24. To be possibly predisposed to cancer makes me angry	0.72					
I20. Possibly being predisposed to cancer doesn’t make sense to me		-0.90				
I18. Being possibly predisposed to cancer is a mystery to me		-0.88				
I19. I don't understand why I might possibly be predisposed to cancer		-0.81				
I17. Being possibly predisposed to cancer is puzzling to me		-0.75				
I21. I have a clear picture or understanding of my possible predisposition to cancer (R)^a^		-0.47				
I14. The negative effects of any cancer predisposition I might have can be prevented by following doctors behavioural advice			0.83			
I15. Behaviours prescribed by the doctors can control my risk of cancer			0.80			
I13. Behaviours prescribed by the doctors will be effective in preventing me from being predisposed to cancer			0.74			
I2. A predisposition to cancer would have major consequences in my life				-0.76		
I6. A predisposition to cancer would cause difficulties for those close to me				-.747		
I5. A predisposition to cancer would have serious financial consequences				-0.66		
I1. A predisposition to cancer is a serious condition.				-0.65		
I3. A predisposition to cancer would not have much effect on my life (R) ^a^				-0.61		
I4. A predisposition to cancer would strongly affect the way others see me				-0.40		
I7. What I do can determine the presence or absence of the risk situations that predispose to cancer					0.81	
I8. Whether I'm in one of the cancer predisposition risk situations depends on me					0.79	
I27. There is a lot which I can do to control the risk situations that predispose to cancer					0.39	
I11. My actions will have no effect on the risk situations that predispose to cancer						0.74
I12. There is very little that can be done to decrease my risk of cancer						0.68
I9. Nothing I do will affect the risk situations that predispose to cancer						0.62
I16. There is nothing which can help to stop me from being predisposed to cancer						0.56
I10. I have the power to influence the risk situations that predispose to cancer (R) ^a^						0.46

^a^ (R) reverse scored items.

*Internal consistency*: The draft instrument demonstrated good internal reliability, with overall Cronbach’s alpha >0.70 (α = 0.82) and those for each of the six domains (emotional, illness coherence, treatment control, consequences, internal control and external control) 0.88, 0.86, 0.74, 0.76, 0.62 and 0.71, respectively. All were good except that for Internal control, which was fair.

#### Divergent validity

The total score of items for measuring each domain of the CPPS was calculated. Higher score of each domain indicates higher negative emotion (Emotional representation), perceived higher consequence of the condition (Consequence), perceived lower illness coherence (Illness coherence), perceived higher Treatment control, External control and Internal control. Table 3 shows that Illness coherence, Treatment control and Internal control were not significantly correlated with CLOT-R, GSES, CHQ-12, PCH and PGH. Emotional representation had low but significant correlations with CLOT-R, CHQ-12 and PCH; Consequence was significantly correlated with CLOT-R, CHQ-12, PCH and PGH while External control was significantly correlated with CLOT-R and GSES. The direction of these correlations are consistent with the measurement intention for the draft instrument, and the low correlations indicate that collinearity is not an issue, providing some support for divergent validity of the draft instrument (Table 2).

**Table 2:** Correlations between scale factors and comparative scales.

	Emotional Representations	Illness Coherence	Treatment Control	Consequences	Internal Control	External Control
CLOT-R	-0.27^a^	-0.05	0.05	-0.17^a^	0.02	-0.19^a^
GSES	-0.11	-0.09	0.09	-0.07	0.08	-0.24^a^
CHQ-12	-0.29^a^	-0.02	-0.04	-0.18^a^	0.04	0.00
PCH	-0.17^a^	-0.08	-0.02	-0.20^a^	0.00	-0.10
PGH	-0.10	-0.04	0.01	-0.18^a^	0.02	-0.07

^a^ Correlation is significant at the 0.01 level (2-tailed).

**Table 3:** Comparison of CPPS domain scores for smokers and passive smokers relative to healthy controls.

Domains	Smokers (M (SD))	Passive smokers (M (SD))	Healthy controls (M (SD))
Emotional representation	14.31 (5.41)	14.71 (5.17)	15.00 (5.59)
Illness coherence	11.88 (3.95)	12.01 (4.00)	11.96 (3.78)
Treatment control	11.17 (2.73)	11.71 (2.18)	11.24 (2.37)
Consequence	18.30 (3.58)	18.03 (4.15)	18.01 (4.26)
Internal control	6.97 (1.98)	6.77 (1.92)	6.82 (1.95)
External control	12.00 (3.54)^a^	11.17 (3.33)	11.02 (3.37)

^a^ p<0.001 after adjustment for age, gender and educational attainment.

M: mean score of a particular CPPS domain; SD: Standard deviation.

#### Discriminant validity

The ability of the scale to discriminate between HBsAg and non-patient groups was indicated within the ‘Treatment Control’ and ‘Consequence’ domains of the CPPS. Compared with the non-patient group, patients perceived higher treatment control (HBsAg group: M = 12.13, SD = 1.59; Non-patient group: M = 11.54, SD = 1.76; t(287) = -2.93, p = 0.004) and higher consequence of cancer predisposition (HBsAg group: M = 21.97, SD = 4.09; Non-patient group: M = 19.85, SD = 4.33; t(287) = -4.25, p<0.001).

### Stage II

#### CFA

**Fig 1:**
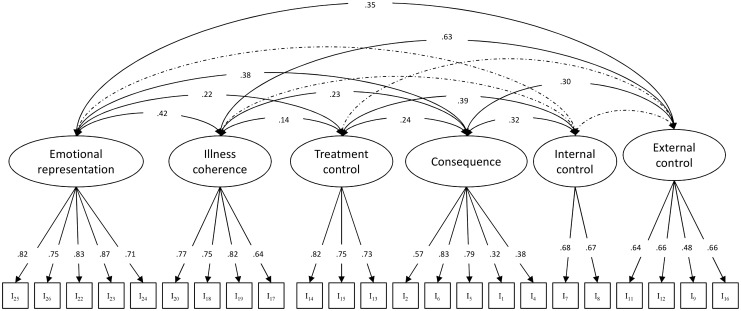
Confirmatory factor analysis for CPPS. Dotted line indicates non-significant correlation between domains. Factor loadings were standardized. All factor loadings and other correlations were statistically significant (p<0.05). Code of each item can be identified in Table 2.

CFA was conducted to test the factor structure derived in Stage I using data of current smokers, passive smokers and community healthy controls. The original model showed poor fit with χ^2^ = 774.33, d_f_ = 284, p<0.001, χ^2^/d_f_ = 2.73, CFI = 0.880, TLI = 0.863, RMSEA = 0.062 (90%CI: 0.057–0.067), SRMR = 0.064. Three items with low standardized factor loadings were identified including one item (I21) on Illness coherence (λ = -0.06), one item (I3) on Consequence (λ = -0.09) and one item (I10) on External control (λ = 0.12). These three items were removed and the CFA was re-run with the remaining 23 items. The revised model showed acceptable fit with χ^2^ = 523.33, d_f_ = 215, p<0.001, χ^2^/d_f_ = 2.43, CFI = 0.921, TLI = 0.907, RMSEA = 0.056 (90%CI: 0.050–0.063), SRMR = 0.047. The standardized factor loading of each indicator and the covariance between CPPS subscales are shown in Fig 1. We also compared this six-factor model with the alternative five-factor model suggested by the IPQ-R [12] to combine the "Internal control" and "External control" as one factor. The resulting data fit for the five-factor model was poor (data not shown).

#### Internal consistency

The internal consistencies of all the six CPPS domains were acceptable with Cronbach's alpha of 0.90, 0.83, 0.81, 0.72, 0.63 and 0.69 for emotional, illness coherence, treatment control, consequences, internal control and external control, respectively.

#### Discriminant validity

The item-sum score of each CPPS domain of smokers and passive smokers were compared against those of the healthy controls. A statistically significant difference was identified between smokers and healthy controls on scores for external control with smokers perceiving higher external control (Table 3). There were no significant differences in scores of other CPPS domains between smokers and healthy controls and in scores of all CPPS domains between passive smokers and healthy controls.

## Discussion

In contrast to the five-factor model describing the parent IPQ-R-GP and other versions of the IPQ-R [12] both the EFA and CFA indicated that a six factor solution for the CPPS best represented this sample of Hong Kong Chinese people. The five core IPQ-R factors are represented as six domains in the CPPS with the additional sixth domain being derived by dividing the IPQ-R ‘personal control’ domain into ‘Internal control’ and ‘External control’. ‘Internal control’ refers to the personal control participants perceived themselves to have over the predisposition while ‘External control’ reflects cancer risk influences that are not amenable to personal control, such as genetics.

The two ‘Internal control’ items contrasted against the five ‘External control’ items are consistent with Rotter’s (1966) proposed internal vs. external locus of control attributes, reflecting the perceived personal malleability of the situation [29]. Responses from these Hong Kong Chinese participants differed for ‘Internal’ and ‘External control’ factor items. The division in the personal control domain in this study may be because many Chinese people hold strongly fatalistic beliefs. The extent to which fatalistic beliefs merge with belief in unchangeable determinants such as genetics is difficult to differentiate but these are clearly separated from controllable risk such as unhealthy diet that increase predisposition. Hence, having the personal control domain split into two types of attributions to personal control makes sense conceptually. The study found that smokers perceived higher external control of their cancer predisposition than non-smokers, indicating that smokers may hold some fatalistic beliefs regarding their addiction to smoking. This may be a barrier for encouraging smoking cessation among smokers.

One item, “To be possibly predisposed to cancer does not worry me” failed to load onto any factors. Worry about cancer predisposition could be either an ‘emotional representation’ or a ‘consequence’. Perceiving no serious consequences from a predisposition would be unlikely to cause worry, whereas, those perceiving a predisposition to be consequential would be more likely to worry about it. Moreover, cancer remains a condition primarily of late adulthood and old age, and for most of these participants, particularly the younger non-patient group, may be subject to complex discounting. The confusion might have resulted in the loading coefficient being too small to for any one factor. The item was eliminated.

The internal consistency of the CPPS was good. All domains except ‘Internal control’ demonstrated good internal consistency. The ‘Internal control’ domain had a moderate alpha of 0.62–0.63 after excluding one item "there is a lot which I can do to control the risk situations that predispose to cancer" but with just two items remaining the scale is brief. One explanation may be that item wordings might be too complex for the participants inducing variation in interpreting the internal control items. The two personal control domains, internal and external control, could be investigated further in the future to better determine how the Chinese population view cancer prevention and control.

The CPPS demonstrated some discriminant validity. HBsAg carriers scored higher on domains of Treatment control and Consequence than did non-patients while smokers scored higher in external control than the healthy controls. HBsAg carriers indicated belief that medical care and advice can minimize their cancer predisposition, supporting the conceptual validity of the measure. HBsAg carriers also considered the consequences of their cancer predisposition to be more serious than did non-patients. Most HBsAg participants recognize their high predisposition of developing liver cancer such and understandably weigh the consequences of this to be more significant than did non-patients. Perceptions of predisposition particularly regarding locus of control could be different for modifiable and unmodifiable cancer risk. There is probably lower perceived predisposition among people who rate their risks as controllable, whereas those with more fatalistic views would rate their predisposition as uncontrollable. Differences in other domain scores were insignificant. This may be partly due to the participants’ characteristics. Some of the non-patient group were relatives of the HBsAg carriers and thereby may feel vulnerable. According to illness-representation theory, coping responses are evoked by individuals’ illness perceptions.^4^ Passive smokers may not perceive themselves differently to those not exposed to second-hand smoke and so appear no different on the CPPS. These effects would dilute differences between groups. Hence, aware HBsAg carriers should more likely seek and adhere to preventive and protective measures than non-patients. The CPPS will enable tests of this prediction.

Domains of CPPS had low though significant correlations with PCH, CCLOT-R, CHQ-12, GSES and PGH. The CPPS was designed to measure how people perceive their predisposition to cancer, and while the correlations were weak, they were in the direction consistent with theoretical expectations for such a construct; namely a more pessimistic and poorer health prospect and greater psychological morbidity.

The study has some limitations. For some participants, item wording may in some instances have been confusing. Refinements to wordings may improve the scale validity. The sample sizes for the present study were limited. Larger samples with different health profiles will provide further information on the validity of the instrument. The instruments in this study were administered individually and completion rate was very high, indicating acceptance by participants. Furthermore, the characteristic differences of the participants between groups may influence the results of discriminant validity tests but we had adjusted the comparisons of CPPS domain scores for major demographic differences including age, gender and educational attainment. The somewhat poor discriminant validity of CPPS may also reflect the risk groups (HBsAg carriers, smokers and passive smokers) being mostly unaware of their cancer predisposition.

In conclusion, the 23-items CPPS demonstrates that the six-factor model can best explain perceptions of cancer predisposition. The scale was found to have preliminarily acceptable construct validity including acceptable internal consistency and divergent validity but only some discriminant validity. Future studies should be conducted to use the construct to predict cancer prevention behaviours in order to test the criterion validity of the construct in different cancer-predisposing risk groups.

## Supporting Information

S1 Dataset S1 Dataset for Stage I. (CSV)

S2 Dataset S2 Dataset for Stage II. (CSV)

S1 Table Respondent’s characteristics, Stage I. (DOCX)

S2 Table Respondents’ characteristics, Stage II. (DOCX)

## Abbreviations

CFA: Confirmatory Factor Analysis
CGSES: The Chinese General Self-Efficacy Scale
CHQ-12: The Chinese Health Questionnaire-12
C-LOT-R: The revised Chinese Life Orientation Test
CPPS: Cancer Predisposition Perception Scale
EFA: exploratory factor analysis
GSES: The General Self-Efficacy Scale
HBV: hepatitis B virus
HBsAg: Hepatitis B surface antigen
HCC: Hepatocellular carcinoma
IPQ: Illness Perception Questionnaire
IPQ-R: Revised Illness Perception Questionnaire
IPQ-R-GP: Revised Illness Perception Questionnaire for genetic predisposition
PCH: Perceived Current Health
PGH: Perceived General Health
