# Supplementary material for: Measuring and Validating a General Cancer Predisposition Perception Scale: An Adaptation of the Revised-IPQ-Genetic Predisposition Scale
Source: PLoS One. 2015 Nov 11;10(11):e0142620. doi: 10.1371/journal.pone.0142620 (PMC4641658; doi:10.1371/journal.pone.0142620)
Supplement: S1 Table — (DOCX) [file pone.0142620.s003.docx]

S1 Table. Participants’ characteristics, Stage I

| Characteristics | HBsAg carriers n = 167 (%) | Healthy controls n = 123 (%) | Differences  (p-value) |
| --- | --- | --- | --- |
| Age (years) Mean (SD) | 45.3 (12.5) | 38.5 (14.9) | 0.001^a^ |
| Gender |  |  |  |
| Male | 93 (55.7) | 38 (31.1) | 0.001^b^ |
| Female | 74 (44.3) | 84 (68.9) |  |
| Martial Status |  |  |  |
| Single | 37 (22.2) | 62 (50.8) | 0.001^b^ |
| Married/cohabiting | 127 (76.0) | 56 (45.9) |  |
| Separated/divorced/ Widowed | 3 (1.8) | 4 (3.2) |  |
| Education |  |  |  |
| None/Primary | 26 (15.6) | 8 (6.6) | <0.001^b^ |
| Secondary | 93 (55.7) | 41 (33.6) |  |
| Tertiary | 48 (28.7) | 73 (59.8) |  |
| Occupation |  |  |  |
| Full-time | 121 (72.5) | 83 (67.5) | 0.415^c^ |
| Part-time | 6 (3.6) | 5 (4.1) |  |
| Retired/Unemployed | 24 (14.4) | 15 (12.2) |  |
| Housewife | 12 (7.2) | 5 (4.1) |  |
| Student | 4 (2.4) | 13 (10.6) |  |
| Missing | 0 (0) | 1 (0.8) |  |

^a^ p-value from t-test; ^b^ p-values from chi-square test; ^c^ p-value from chi-square test based on whether the participants had a full-time job or not.
