# Supplementary material for: Measuring and Validating a General Cancer Predisposition Perception Scale: An Adaptation of the Revised-IPQ-Genetic Predisposition Scale
Source: PLoS One. 2015 Nov 11;10(11):e0142620. doi: 10.1371/journal.pone.0142620 (PMC4641658; doi:10.1371/journal.pone.0142620)
Supplement: S2 Table — (DOCX) [file pone.0142620.s004.docx]

S2 Table. Participants’ characteristics, Stage II

|  | Controls (N=148) | Current smokers (N=150) | Passive smokers (N=152) | Differences  (p-value)^a^ |
| --- | --- | --- | --- | --- |
| Gender (female) | 91 (60.8%) | 41 (27.3%) | 121 (79.6%) | <0.001 |
| Age (years) |  |  |  |  |
| 18-34 | 39 (26.2%) | 22 (14.7%) | 41 (27.0%) | 0.001 |
| 35-54 | 45 (30.2%) | 76 (50.7%) | 65 (42.8%) |  |
| ≥55 | 65 (43.6%) | 52 (34.6%) | 46 (30.3%) |  |
| Mean (years) | 47.4 | 49.4 | 44.9 |  |
| Marital status |  |  |  |  |
| Single | 40 (26.8%) | 31 (20.7%) | 40 (26.3%) | 0.376 |
| Married/ cohabited/ formerly married | 109 (73.1%) | 119 (79.3%) | 112 (73.7%) |  |
| Education |  |  |  |  |
| ≤Primary | 27 (18.2%) | 30 (20.0%) | 23 (15.1%) | 0.019 |
| Secondary | 65 (43.9%) | 90 (60.0%) | 88 (57.9%) |  |
| ≥Tertiary | 56 (37.8%) | 30 (20.0%) | 41 (27.0%) |  |
| Occupation |  |  |  |  |
| Full-time | 58 (39.5%) | 85 (57.0%) | 51 (33.5%) | <0.001 |
| Part-time/ Retired/ housewife/ students /unemployed | 89 (60.5%) | 64 (42.9%) | 101 (66.4%) |  |

^a^ p-values based on chi-square test.
